# Ethyl 1-[(2-chloro-1,3-thia­zol-5-yl)methyl]-5-methyl-1*H*-1,2,3-triazole-4-carboxylate

**DOI:** 10.1107/S1600536808037914

**Published:** 2008-11-20

**Authors:** Xiao-Bao Chen, Feng-Mei Sun, Jing Xu, Zuan Ma, Ai-Hua Zheng

**Affiliations:** a Department of Medicinal Chemistry, Yunyang Medical College, Shiyan, Hubei 442000, People’s Republic of China; b School of Chemistry and Chemical Engineering, Henan Institute of Science and Technology, Xinxiang, Henan 453003, People’s Republic of China

## Abstract

In the title compound, C_10_H_11_ClN_4_O_2_S, the triazole ring carries methyl and ethoxy­carbonyl groups and is bound *via* a methyl­ene bridge to a chloro­thia­zole unit. There is also evidence for significant electron delocalization in the triazolyl system. Intra- and inter­molecular C—H⋯O hydrogen bonds together with strong π–π stacking inter­actions [centroid–centroid distance 3.620 (1) Å] stabilize the structure.

## Related literature

Many derivatives of triazole have been prepared, and their biological activities have been studied by Ogura *et al.* (2000 ▶), Najim *et al.* (2004 ▶), Abu-Orabi *et al.* (1989 ▶), Shuto *et al.* (1995 ▶), Fan & Katritsky (1996 ▶), Chen *et al.* (2005 ▶) and Liu *et al.* (2001 ▶). For the synthesis, see: Chen *et al.* (2007 ▶); Chen & Shi (2008 ▶). For bond-length data, see: Sasada (1984 ▶); Wang *et al.* (1998 ▶). For related literature, see: Chen *et al.* (2007 ▶); Tian *et al.* (2008 ▶); Chen *et al.* (2008 ▶); Knox & Rogers (1989 ▶); Rogers *et al.* (1985 ▶); Shuto *et al.* (1995 ▶).
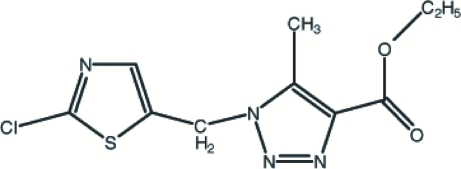


## Experimental

### 

#### Crystal data

C_10_H_11_ClN_4_O_2_S
*M* *_r_* = 286.74
Triclinic, 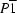

*a* = 7.9692 (14) Å
*b* = 9.1656 (16) Å
*c* = 10.4430 (18) Å
α = 65.892 (2)°
β = 67.938 (2)°
γ = 80.641 (2)°
*V* = 645.23 (19) Å^3^
*Z* = 2
Mo *K*α radiation
μ = 0.46 mm^−1^
*T* = 291 (2) K
0.50 × 0.40 × 0.30 mm

#### Data collection

Bruker SMART APEX CCD area-detector diffractometer
Absorption correction: none
4630 measured reflections
2332 independent reflections
2005 reflections with *I* > 2σ(*I*)
*R*_int_ = 0.018

#### Refinement

*R*[*F* ^2^ > 2σ(*F* ^2^)] = 0.041
*wR*(*F* ^2^) = 0.118
*S* = 1.04
2332 reflections
165 parameters
H-atom parameters constrained
Δρ_max_ = 0.30 e Å^−3^
Δρ_min_ = −0.25 e Å^−3^

### 

Data collection: *SMART* (Bruker, 2000 ▶); cell refinement: *SAINT* (Bruker, 2000 ▶); data reduction: *SAINT*; program(s) used to solve structure: *SHELXS97* (Sheldrick, 2008 ▶); program(s) used to refine structure: *SHELXL97* (Sheldrick, 2008 ▶); molecular graphics: *SHELXTL* (Sheldrick, 2008 ▶); software used to prepare material for publication: *SHELXTL*.

## Supplementary Material

Crystal structure: contains datablocks global, I. DOI: 10.1107/S1600536808037914/at2677sup1.cif

Structure factors: contains datablocks I. DOI: 10.1107/S1600536808037914/at2677Isup2.hkl

Additional supplementary materials:  crystallographic information; 3D view; checkCIF report

## Figures and Tables

**Table 1:** Hydrogen-bond geometry (Å, °)

*D*—H⋯*A*	*D*—H	H⋯*A*	*D*⋯*A*	*D*—H⋯*A*
C2—H2⋯O1^i^	0.93	2.47	3.375 (4)	164
C7—H7*B*⋯O2	0.96	2.43	3.033 (4)	121

Symmetry code: (i) 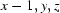.

**Fig. 1.**
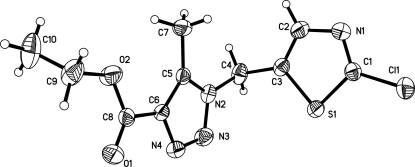
View of the molecular structure of (I), showing the atom labelling scheme and with displacement ellipsoids drawn at the 50% probability level.

**Fig. 2.**
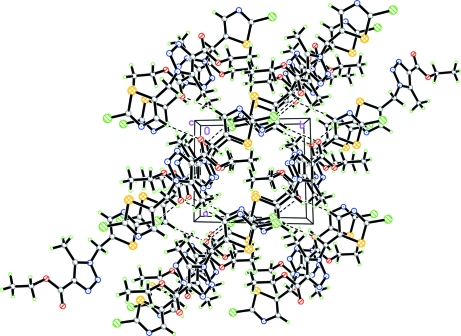
A partial view of the crystal packing of (I), showing the formation of C—H···O hydrogen-bonding interactions (dashed lines).

## supplementary crystallographic information

### Comment

It is well known that many triazole-related molecules play an important role in the development of agrochemicals such as insecticides, nematocides, acaricide and plant growth regulators (Ogura *et al.*, 2000; Najim *et al.*, 2004; Abu-Orabi *et al.*, 1989; Shuto *et al.*,1995; Fan & Katritsky, 1996; Chen *et al.*, 2005; Richard & Ben, 1985; Ingrid *et al., *1989 and Liu *et al.*, 2001). Since the structure-activity relationship is very useful in the rational design of pharmaceuticals and agrochemicals. We report here the crystal structure of the title compound, (I) (Fig. 1), which was synthesized by introducing pyridine rings into a 1,2,3-triazole molecular framework.

In the title compound, the C5—N2 and C6—N4 bonds are significantly shorter than that of the single bond of C—N (1.47 Å; Sasada, 1984) and close to the value of the double bond of C—N (1.28 Å; Wang *et al.*, 1998). This indicates significant electron delocalization in the triazolyl system.

Inter and intramolecular C—H···O hydrogen bonds contribute strongly to the stability of the molecular configuration (Fig.2). Strong π—π stacking interactions are also found between adjacent S1—C1/N1/C2—C3 rings with centroid-centroid distances 3.620 (1) Å, dihedral angles of 0.03 (1)°, and a shortest interplanar distance of 3.573 Å.

### Experimental

Ethyl acetylacetate (2 mmol) and 5-azidomethyl-2-chlorothiazole (2 mmol) were added to a suspension of milled potassium carbonate (2 mmol) in DMSO (10 ml). The mixture was stirred at room temperature for 6 h (monitored by thin-layer chromatography) and poured to water (50 ml). The solid was collected by filtration, washed with water and diethyl ether, respectively, and dried to give 0.52 g of the title compound (yield 91%). Colourless crystals of (I) suitable for X-ray structure analysis were grown from acetone and petroleum ether (1:1, *v*/*v*).

### Refinement

H atoms bonded to C were placed at calculated positions, with C—H distances in the range 0.93 - 0.98Å. They were refined using a riding model, with *U*_iso_(H) = 1.2*U*_eq_(C), or 1.5*U*_eq_(methyl C).

### Crystal data

**Table:**

C_10_H_11_ClN_4_O_2_S	*Z* = 2
*M**_r_* = 286.74	*F*_000_ = 296
Triclinic, *P*1	*D*_x_ = 1.476 Mg m^−^^3^
Hall symbol: -P 1	Mo *K*α radiation λ = 0.71073 Å
*a* = 7.9692 (14) Å	Cell parameters from 2592 reflections
*b* = 9.1656 (16) Å	θ = 2.4–27.4º
*c* = 10.4430 (18) Å	µ = 0.46 mm^−^^1^
α = 65.892 (2)º	*T* = 291 (2) K
β = 67.938 (2)º	Block, colourless
γ = 80.641 (2)º	0.50 × 0.40 × 0.30 mm
*V* = 645.23 (19) Å^3^	

### Data collection

**Table:**

Bruker SMART APEX CCD area-detector diffractometer	2005 reflections with *I* > 2σ(*I*)
Radiation source: fine-focus sealed tube	*R*_int_ = 0.018
Monochromator: graphite	θ_max_ = 25.5º
*T* = 291(2) K	θ_min_ = 2.4º
φ and ω scans	*h* = −9→9
Absorption correction: none	*k* = −11→11
4630 measured reflections	*l* = −12→12
2332 independent reflections	

### Refinement

**Table:**

Refinement on *F*^2^	Secondary atom site location: difference Fourier map
Least-squares matrix: full	Hydrogen site location: inferred from neighbouring sites
*R*[*F*^2^ > 2σ(*F*^2^)] = 0.041	H-atom parameters constrained
*wR*(*F*^2^) = 0.118	*w* = 1/[σ^2^(*F*_o_^2^) + (0.062*P*)^2^ + 0.2829*P*] where *P* = (*F*_o_^2^ + 2*F*_c_^2^)/3
*S* = 1.04	(Δ/σ)_max_ < 0.001
2332 reflections	Δρ_max_ = 0.30 e Å^−^^3^
165 parameters	Δρ_min_ = −0.25 e Å^−^^3^
Primary atom site location: structure-invariant direct methods	Extinction correction: none

### Special details

**Table:**

Geometry. All e.s.d.'s (except the e.s.d. in the dihedral angle between two l.s. planes)are estimated using the full covariance matrix. The cell e.s.d.'s are takeninto account individually in the estimation of e.s.d.'s in distances, anglesand torsion angles; correlations between e.s.d.'s in cell parameters are onlyused when they are defined by crystal symmetry. An approximate (isotropic)treatment of cell e.s.d.'s is used for estimating e.s.d.'s involving l.s. planes.
Refinement. Refinement of *F*^2^ against ALL reflections. The weighted *R*-factor *wR* andgoodness of fit *S* are based on *F*^2^, conventional *R*-factors *R* are basedon *F*, with *F* set to zero for negative *F*^2^. The threshold expression of*F*^2^ > σ(*F*^2^) is used only for calculating *R*-factors(gt) *etc*. and isnot relevant to the choice of reflections for refinement. *R*-factors basedon *F*^2^ are statistically about twice as large as those based on *F*, and *R*-factors based on ALL data will be even larger.

### Fractional atomic coordinates and isotropic or equivalent isotropic displacement parameters (Å^2^)

**Table:**

	*x*	*y*	*z*	*U*_iso_*/*U*_eq_	
Cl1	−0.01266 (10)	0.69136 (8)	0.20124 (9)	0.0704 (2)	
S1	0.23526 (8)	0.46808 (7)	0.07021 (7)	0.0534 (2)	
O1	0.7825 (3)	−0.0891 (2)	0.3892 (2)	0.0715 (5)	
O2	0.5259 (3)	−0.2272 (2)	0.5002 (2)	0.0718 (6)	
N1	−0.0923 (3)	0.4021 (3)	0.2475 (3)	0.0674 (6)	
N2	0.4074 (3)	0.0929 (2)	0.1275 (2)	0.0486 (5)	
N3	0.5752 (3)	0.1573 (3)	0.0531 (2)	0.0603 (5)	
N4	0.6709 (3)	0.0880 (2)	0.1400 (2)	0.0567 (5)	
C1	0.0281 (3)	0.5109 (3)	0.1813 (3)	0.0496 (5)	
C2	−0.0196 (4)	0.2725 (3)	0.2086 (3)	0.0676 (7)	
H2	−0.0873	0.1816	0.2446	0.081*	
C3	0.1528 (3)	0.2836 (3)	0.1165 (3)	0.0479 (5)	
C4	0.2660 (4)	0.1603 (3)	0.0601 (3)	0.0584 (6)	
H4A	0.1887	0.0750	0.0830	0.070*	
H4B	0.3222	0.2087	−0.0476	0.070*	
C5	0.3935 (3)	−0.0189 (2)	0.2639 (2)	0.0419 (5)	
C6	0.5634 (3)	−0.0210 (2)	0.2704 (2)	0.0435 (5)	
C7	0.2239 (3)	−0.1066 (3)	0.3707 (3)	0.0611 (7)	
H7A	0.1451	−0.0411	0.4214	0.092*	
H7B	0.2521	−0.2038	0.4424	0.092*	
H7C	0.1648	−0.1314	0.3172	0.092*	
C8	0.6388 (3)	−0.1135 (3)	0.3903 (3)	0.0472 (5)	
C9	0.5797 (5)	−0.3295 (3)	0.6289 (4)	0.0808 (9)	
H9A	0.6961	−0.2958	0.6163	0.097*	
H9B	0.4914	−0.3195	0.7191	0.097*	
C10	0.5916 (6)	−0.4920 (4)	0.6429 (4)	0.1001 (13)	
H10A	0.4813	−0.5209	0.6427	0.150*	
H10B	0.6104	−0.5599	0.7349	0.150*	
H10C	0.6913	−0.5045	0.5605	0.150*	

### Atomic displacement parameters (Å^2^)

**Table:**

	*U*^11^	*U*^22^	*U*^33^	*U*^12^	*U*^13^	*U*^23^
Cl1	0.0673 (4)	0.0627 (4)	0.0875 (5)	0.0081 (3)	−0.0224 (4)	−0.0417 (4)
S1	0.0525 (4)	0.0453 (3)	0.0548 (4)	−0.0034 (3)	−0.0102 (3)	−0.0180 (3)
O1	0.0572 (11)	0.0679 (12)	0.0968 (14)	−0.0051 (9)	−0.0423 (10)	−0.0223 (10)
O2	0.0735 (12)	0.0639 (11)	0.0695 (11)	−0.0188 (9)	−0.0428 (10)	0.0060 (9)
N1	0.0479 (12)	0.0600 (13)	0.0824 (15)	−0.0036 (10)	−0.0144 (11)	−0.0218 (12)
N2	0.0597 (12)	0.0394 (9)	0.0494 (10)	0.0052 (9)	−0.0230 (9)	−0.0176 (8)
N3	0.0638 (13)	0.0530 (12)	0.0515 (11)	−0.0068 (10)	−0.0123 (10)	−0.0125 (9)
N4	0.0518 (11)	0.0514 (11)	0.0573 (12)	−0.0067 (9)	−0.0114 (9)	−0.0157 (10)
C1	0.0496 (12)	0.0485 (12)	0.0504 (12)	0.0045 (10)	−0.0207 (10)	−0.0174 (10)
C2	0.0589 (16)	0.0488 (14)	0.092 (2)	−0.0081 (12)	−0.0286 (14)	−0.0188 (14)
C3	0.0576 (14)	0.0418 (11)	0.0486 (12)	0.0006 (10)	−0.0291 (11)	−0.0117 (10)
C4	0.0796 (17)	0.0483 (13)	0.0613 (15)	0.0104 (12)	−0.0412 (13)	−0.0229 (12)
C5	0.0455 (11)	0.0347 (10)	0.0480 (11)	0.0035 (9)	−0.0177 (9)	−0.0182 (9)
C6	0.0433 (11)	0.0354 (10)	0.0499 (12)	−0.0012 (9)	−0.0128 (9)	−0.0171 (9)
C7	0.0465 (13)	0.0571 (15)	0.0709 (16)	−0.0079 (11)	−0.0228 (12)	−0.0108 (12)
C8	0.0464 (12)	0.0397 (11)	0.0621 (14)	0.0046 (10)	−0.0221 (10)	−0.0243 (10)
C9	0.109 (2)	0.0618 (17)	0.0772 (19)	−0.0068 (17)	−0.0621 (19)	−0.0026 (15)
C10	0.162 (4)	0.068 (2)	0.098 (2)	0.034 (2)	−0.087 (3)	−0.0328 (18)

### Geometric parameters (Å, °)

**Table:**

Cl1—C1	1.715 (2)	C3—C4	1.501 (3)
S1—C1	1.717 (2)	C4—H4A	0.9700
S1—C3	1.726 (2)	C4—H4B	0.9700
O1—C8	1.197 (3)	C5—C6	1.378 (3)
O2—C8	1.328 (3)	C5—C7	1.485 (3)
O2—C9	1.464 (3)	C6—C8	1.476 (3)
N1—C1	1.281 (3)	C7—H7A	0.9600
N1—C2	1.380 (4)	C7—H7B	0.9600
N2—C5	1.349 (3)	C7—H7C	0.9600
N2—N3	1.357 (3)	C9—C10	1.427 (5)
N2—C4	1.470 (3)	C9—H9A	0.9700
N3—N4	1.304 (3)	C9—H9B	0.9700
N4—C6	1.370 (3)	C10—H10A	0.9600
C2—C3	1.340 (4)	C10—H10B	0.9600
C2—H2	0.9300	C10—H10C	0.9600
			
C1—S1—C3	88.37 (12)	C6—C5—C7	133.6 (2)
C8—O2—C9	118.2 (2)	N4—C6—C5	109.60 (19)
C1—N1—C2	108.7 (2)	N4—C6—C8	119.0 (2)
C5—N2—N3	111.70 (19)	C5—C6—C8	131.4 (2)
C5—N2—C4	129.3 (2)	C5—C7—H7A	109.5
N3—N2—C4	118.8 (2)	C5—C7—H7B	109.5
N4—N3—N2	107.38 (18)	H7A—C7—H7B	109.5
N3—N4—C6	108.2 (2)	C5—C7—H7C	109.5
N1—C1—Cl1	122.4 (2)	H7A—C7—H7C	109.5
N1—C1—S1	116.79 (19)	H7B—C7—H7C	109.5
Cl1—C1—S1	120.83 (14)	O1—C8—O2	124.3 (2)
C3—C2—N1	117.0 (2)	O1—C8—C6	124.5 (2)
C3—C2—H2	121.5	O2—C8—C6	111.18 (18)
N1—C2—H2	121.5	C10—C9—O2	110.0 (3)
C2—C3—C4	128.2 (2)	C10—C9—H9A	109.7
C2—C3—S1	109.13 (19)	O2—C9—H9A	109.7
C4—C3—S1	122.70 (19)	C10—C9—H9B	109.7
N2—C4—C3	111.66 (18)	O2—C9—H9B	109.7
N2—C4—H4A	109.3	H9A—C9—H9B	108.2
C3—C4—H4A	109.3	C9—C10—H10A	109.5
N2—C4—H4B	109.3	C9—C10—H10B	109.5
C3—C4—H4B	109.3	H10A—C10—H10B	109.5
H4A—C4—H4B	107.9	C9—C10—H10C	109.5
N2—C5—C6	103.16 (19)	H10A—C10—H10C	109.5
N2—C5—C7	123.2 (2)	H10B—C10—H10C	109.5
			
C5—N2—N3—N4	0.1 (3)	C4—N2—C5—C6	−173.8 (2)
C4—N2—N3—N4	174.52 (19)	N3—N2—C5—C7	178.8 (2)
N2—N3—N4—C6	−0.1 (3)	C4—N2—C5—C7	5.1 (3)
C2—N1—C1—Cl1	179.52 (19)	N3—N4—C6—C5	0.0 (3)
C2—N1—C1—S1	−0.5 (3)	N3—N4—C6—C8	−178.4 (2)
C3—S1—C1—N1	0.1 (2)	N2—C5—C6—N4	0.0 (2)
C3—S1—C1—Cl1	−179.93 (15)	C7—C5—C6—N4	−178.6 (2)
C1—N1—C2—C3	0.8 (4)	N2—C5—C6—C8	178.2 (2)
N1—C2—C3—C4	178.2 (2)	C7—C5—C6—C8	−0.4 (4)
N1—C2—C3—S1	−0.8 (3)	C9—O2—C8—O1	0.2 (4)
C1—S1—C3—C2	0.38 (19)	C9—O2—C8—C6	−179.5 (2)
C1—S1—C3—C4	−178.66 (19)	N4—C6—C8—O1	8.3 (3)
C5—N2—C4—C3	78.7 (3)	C5—C6—C8—O1	−169.7 (2)
N3—N2—C4—C3	−94.6 (3)	N4—C6—C8—O2	−171.9 (2)
C2—C3—C4—N2	−109.0 (3)	C5—C6—C8—O2	10.0 (3)
S1—C3—C4—N2	69.9 (3)	C8—O2—C9—C10	−119.0 (3)
N3—N2—C5—C6	−0.1 (2)		

### Hydrogen-bond geometry (Å, °)

**Table:**

*D*—H···*A*	*D*—H	H···*A*	*D*···*A*	*D*—H···*A*
C2—H2···O1^i^	0.93	2.47	3.375 (4)	164
C7—H7B···O2	0.96	2.43	3.033 (4)	121
C9—H9A···O1	0.97	2.28	2.710 (4)	106

Symmetry codes: (i) *x*−1, *y*, *z*.
